# Determinants of survival and recurrence in patients with stage I colorectal cancer

**DOI:** 10.3389/fsurg.2024.1377733

**Published:** 2024-05-15

**Authors:** Alimohammad Bananzadeh, Mahshid Bahadori, Mohammad Mohammadianpanah, Faramarz Pakravan, Sara Shojaei-Zarghani, Ali Reza Safarpour

**Affiliations:** ^1^ Laparoscopy Research Center, Shiraz University of Medical Sciences, Shiraz, Iran; ^2^ Colorectal Research Center, Shiraz University of Medical Sciences, Shiraz, Iran; ^3^ Private Practice for Pelvic Floor, Continence Disorders and Coloproctology, Düsseldorf, Germany

**Keywords:** colorectal neoplasms, recurrence, survival, cohort studies, neoplasm metastasis

## Abstract

**Background:**

Due to the novel advanced screening methods, the number of patients diagnosed with stage I colorectal cancer (CRC) is increasing. This retrospective cohort study aimed to identify recurrence and survival risk factors of patients with stage I CRC after surgery.

**Materials and methods:**

Patients with stage I CRC were evaluated, and their demographic and clinicopathologic variables were recorded. The log-rank test assessed the association of variables with overall survival (OS), recurrence-free survival (RFS), local recurrence, and distant metastasis.

**Results:**

The median overall survival period was 51 months. The recurrence rate was 13.7%: 7.2% local and 9.3% distant recurrence. One-, two-, three-, and five-year RFS were 92%, 89%, 87%, and 83%, respectively, and OS were 96%, 93%, 90%, and 89%, respectively. Local and distant recurrence rates were higher in patients with higher tumor grades. Additionally, RFS and OS were worse in patients with higher tumor grades, and perforation was associated with worse OS.

**Conclusions:**

The determinants of survival and recurrence identified in the present study can be used to improve patient outcomes by early diagnosis and appropriate management of high-risk patients.

## 1 Introduction

Colorectal cancer (CRC) is the third most common cancer and the second cause of cancer-related death worldwide (1), with an increasing trend of incidence, especially in countries with low to medium human development indices (2). Recent national cancer registry reports in Iran indicate CRC as the third most common cancer (3), previously the fourth, with an increasing trend in its incidence, attributed to the growing use of screening programs and Western lifestyles (4, 5). Recent advances in cancer screening programs and novel management strategies have resulted in an overall reduced mortality of CRC (6). The overall survival (OS) rate of CRC has also improved in recent years in Iran; however, due to varying results across different regions, further studies are required about the five-year OS rate and its predictors (7, 8).

Several factors have been identified as prognostic factors of CRC, including patient-related factors, such as age at diagnosis, sex, family history, and ethnicity, and tumor-related factors, such as tumor, node, and metastasis (TNM) staging, tumor grade, histologic type, depth of invasion, and perineural invasion (9, 10). As higher TNM stages have a poorer prognosis, research has focused on novel strategies of diagnosis and treatment for high-stage tumors, the results of which have improved the OS of patients with CRC stages II and III during the past few years; however, the OS of stage I CRC has remained stable, and less attention has been directed toward this stage of the disease (11).

Stage I CRC involves growth through the mucosa with invasion into the muscular layer without metastasis to nearby tissues or lymph nodes (T1 or T2, N0, M0) (12). Improved screening for CRC has resulted in the identification of more tumors at lower stages (13, 14); accordingly, with the increased incidence of stage I CRC, trends of CRC recurrence may also alter (15). Segmental resection is the current treatment of choice for stage I CRC (16), but with the change in the trend of incidence and recurrence, a change in management strategies may also be required. However, studies have not separated survival and recurrence rates based on the tumor stage (17, 18) or have reported the results of early or localized stage tumors together (19). Therefore, updated reports are required for the rates and predictors of OS and recurrence in stage I CRC. Furthermore, given that the incidence of CRC in Iran varies depending on the country's geographical region, it is imperative to conduct separate studies on CRC in each province. These variations may be attributed to disparities in genetic, ethnic, and demographic characteristics (such as age and sex distributions), socioeconomic status, lifestyle habits, access to healthcare services and therapeutic approaches, as well as industrial status that impact air, water, and soil conditions across different provinces of Iran (8). Therefore, the present study aimed to evaluate clinicopathological characteristics and oncologic outcomes of patients with stage I CRC, delineating the rates and predictors of recurrence and survival five years after surgery.

## 2 Materials and methods

All patients who underwent curative surgery for stage I CRC at the Shahid Faghihi or Nemazee Hospital (Shiraz, Iran) from 2005 to 2014 were considered the study population. The protocol of this retrospective cohort study was approved by the Ethics Committee of Shiraz University of Medical Sciences (code: IR.SUMS.MED.REC.1399.171). The included patients signed a written informed consent upon referral explaining that their deidentified data might be used in future research.

Histopathologic studies confirmed the diagnosis of CRC, and all patients required surgery. Patients who received neoadjuvant chemotherapy before surgery or had other malignancies were excluded. Furthermore, patients with tumor-positive margin or who experienced postoperative complications were excluded from the study. All patients with the inclusion criteria were enrolled in the study by census method.

We recorded demographic (sex and age) and clinicopathologic variables, including tumor size, location, grade, histology, T stage, lymphovascular invasion (LVI), perineural invasion (PNI), number of lymph nodes, surgical approach and technique, and gross findings (such as obstruction, perforation, and appearance). The tumor site was categorized as right colon (including ileocecal valve, cecum, ascending colon, and hepatic flexure), left colon (splenic flexure, descending colon, and sigmoid), and transverse colon. The rectum was defined as the last 15 cm of the gastrointestinal tract proximal to the anal canal. An experienced pathologist performed the histopathologic evaluation of the tumor site, size, LVI, PNI, and number of lymph nodes. TNM staging was done per the American Joint Cancer Committee (20).

The patients were visited by the radio-oncologist once every three months for the first two years after surgery, then every six months for 3–5 years, and then every year. At each visit, patients underwent physical examination by the physician. Serum levels of carcinoembryonic antigen (CEA) were checked every six months, while computed tomography (CT) scans of the chest, abdomen, and pelvis were requested annually. Chest x-ray and abdominal ultrasound examination were used as an alternative if CT was unavailable.

The main outcomes included overall survival (OS), recurrence-free survival (RFS), and local and distant recurrence. OS was considered the first day after surgery until the last follow-up or death.

Patients who failed to refer for follow-up were excluded from the study.

### 2.1 Statistical analysis

Results are presented as mean ± standard deviation (SD) for quantitative variables and as frequency (percentage) for categorical variables. The one-sample Kolmogorov-Smirnov test was used to determine the normal distribution of data, and Levene's test was used to test the equality of variances. Continuous variables were compared using the *t-*test or Mann-Whitney *U*-test whenever the data did not appear to have normal distribution or when the assumption of equal variances was violated across the study groups. Categorical variables were compared using the chi-squared or Fisher's exact test based on the number of patients. The association of each variable with OS and RFS was evaluated using univariate analysis performed by the log-rank test. Considering the results of the Kaplan-Meier analysis, variables with *P*-values ≤0.1 as well as age and sex entered the Cox regression model for estimating the predictors of OS and RFS by multivariate analysis; we used the step-by-step backward conditional method based on the conditional likelihood ratio. IBM SPSS Statistics for Windows version 21.0 (IBM Corp. 2012, Armonk, NY: IBM Corp.) was used for the statistical analysis. For the statistical significance, *P*-values <0.05 were considered significant.

## 3 Results

The demographic and clinicopathological characteristics are shown in Table 1. Of 295, 159 were men (54.6%); 26 patients were <40 years old (8.9%), 134 were 40–60 years old (46.0%), and 131 were ≥60 years old (45.0%). The median overall survival period was 51 months (interquartile range: 25–77 months). The mean age of men was significantly higher than that of women (58.2 ± 12.9 vs. 55.3 ± 12.2 years, respectively; *P* = 0.03). The tumor site was the colon in 123 cases (50 right colon and 73 left colon) and rectum in 168 cases (57.7%). Tumor grade I was observed in 226 patients (77.7%), grade II in 48 patients (16.5%), and grade III in 8 cases (2.7%; 9 cases missing); 43 patients T1 and 235 patients T2 (13 cases missing). Eighteen cases had LVI (6.2%) and 6 had PNI (2.1%). The number of extracted lymph nodes was <5 in 127 patients (43.6%), 5–12 in 110 patients (37.8%), and ≥12 in 54 patients (18.5%). The histologic type did not vary according to patients' sex (*P* = 0.859) or mean age at diagnosis (*P* = 0.403).

**Table 1:** The difference in local and distant recurrence based on the study variables.

Variables	Variables levels	Total (*N* = 291)	Local recurrence (*N* = 21)	*P*-value	Distant metastasis (*N* = 27)	*P*-value
Sex, *n* (%)	Male	159 (54.6)	12 (7.5)	0.811*	13 (8.2)	0.477*
	Female	132 (45.4)	9 (6.8)		14 (10.6)	
Age (years), *n* (%)	<40	26 (8.9)	3 (11.5)	0.671*	3 (11.5)	0.858*
	40–60	134 (46.0)	9 (6.7)		13 (9.7)	
	≥60	131 (45.0)	9 (6.9)		11 (8.4)	
Tumor size (cm), *n* (%)	<3	113 (38.8)	11 (9.7)	0.375*	15 (13.3)	0.148*
	3–5	103 (35.4)	7 (6.8)		9 (8.7)	
	≥5	53 (18.2)	2 (3.8)		2 (3.8)	
Tumor location, *n* (%)	Colon	123 (42.3)	6 (4.9)	0.187*	10 (8.1)	0.563*
	Rectum	168 (57.7)	15 (8.9)		17 (10.1)	
Tumor laterality, *n* (%)	Right colon	50 (17.2)	4 (8.0)	0.227*	5 (10.0)	0.710*
	Left colon	73 (25.1)	2 (2.7)		5 (6.8)	
	Rectum	168 (57.7)	15 (8.9)		17 (10.1)	
Tumor grade, *n* (%)	I	226 (77.7)	13 (5.8)	0.043*	18 (8.0)	<0.001*
	II	48 (16.5)	6 (12.5)		5 (10.4)	
	III	8 (2.7)	2 (25.0)		4 (50.0)	
T level, *n* (%)	T1	43 (14.8)	4 (9.3)	0.545**	2 (4.7)	0.276**
	T2	235 (80.8)	17 (7.2)		25 (10.6)	
Perineural invasion, *n* (%)	Yes	6 (2.1)	1 (16.7)	0.365**	1 (16.7)	0.445**
	No	285 (97.9)	20 (7.0)		26 (9.1)	
Tumor appearance, *n* (%)	Fungative or polypoid	57 (19.6)	4 (7.0)	0.173**	6 (10.5)	0.941*
	Ulcerative	56 (19.2)	3 (5.4)		8 (14.3)	
	Ulcerative fungative	16 (5.5)	3 (18.8)		2 (12.5)	
	Infiltrative	18 (6.2)	3 (16.7)		2 (11.1)	
Obstruction, *n* (%)	Yes	7 (2.4)	0 (0.0)	>0.999**	1 (14.3)	0.498**
	No	284 (97.6)	21 (7.4)		26 (9.2)	
Perforation, *n* (%)	Yes	8 (2.7)	0 (0.0)	>0.999**	1 (12.5)	0.546**
	No	283 (97.2)	21 (7.4)		26 (9.2)	
Surgical Approach, *n* (%)	Open	92 (31.6)	8 (8.7)	0.410*	8 (8.7)	0.806*
	Laparoscopy	198 (68.0)	12 (6.1)		19 (9.6)	

*P*-value <0.05 was considered significant.

* The results of chi square test.

** The result of Fisher's exact test.

Bold values represent significant results.

### 3.1 Recurrence

Of all patients, 40 experienced CRC recurrence (13.7%): 7.2% local and 9.3% distant metastases. The difference in the status of recurrence according to the study variables is shown in Table 1. Local and distant recurrence did not differ according to patients' sex, age, tumor size, site, laterality, T stage, PNI, histologic type, surgical approach, obstruction, and perforation. Patients with positive LVI had higher distant metastasis rates (*P* = 0.005), but not local recurrence (*P* = 0.131). Local and distant recurrence varied depending on the tumor grade (*P* = 0.043 and *P* < 0.001, respectively, Table 1).

### 3.2 Survival rate

**Figure 1:**
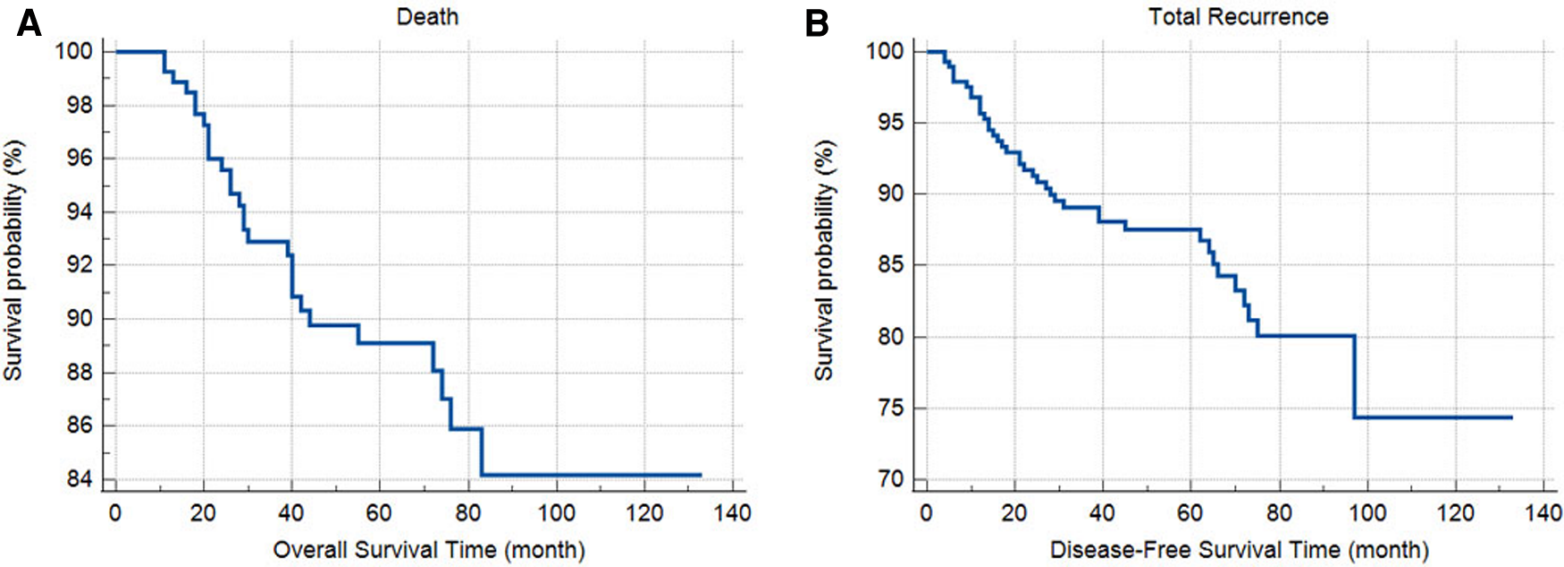
The overall (**A**) and recurrence-free (**B**) survival of patients.

One-, two-, three-, and five-year RFS were 92%, 89%, 87%, and 83%, respectively, and OS were 96%, 93%, 90%, and 89%, respectively. Variations in RFS and OS based on the study variables are shown in Table 2. RFS and OS rates did not differ according to the patients' sex, age categories, tumor size, site, laterality, T stage, PNI, LVI, histologic type, tumor appearance, and obstruction. However, RFS and OS rates were higher in patients with lower tumor grade compared to the higher (*P* < 0.001 and 0.001, respectively) (Table 2). Furthermore, OS was lower in patients with perforation compared to the others (*P* = 0.020). The overall and recurrence-free survival rates of patients within five years are depicted in Figure 1.

**Table 2:** The univariate analysis for all potential prognostic variables in overall and recurrence-free survival .

Variables	Levels	Recurrence-free survival (*n* = 251)	*P*-value*	Overall survival (*n* = 263)	*P*-value*
Sex, *n* (%)	Male	139 (87.4)	0.737	143 (89.9)	0.605
	Female	112 (84.8)		120 (90.9)	
Age categories, *n* (%)	<40	21 (80.8)	0.812	23 (88.5)	0.118
	40–60	117 (87.3)		126 (94.0)	
	≥60	113 (86.3)		114 (87.0)	
Tumor size, *n* (%)	<3	60 (88.2)	0.786	64 (88.9)	0.740
	3–5	84 (88.4)		95 (89.6)	
	≥5	83 (90.2)		85 (90.4)	
Tumor location, *n* (%)	Colon	109 (88.6)	0.452	113 (91.9)	0.568
	Rectum	142 (84.5)		150 (89.3)	
Tumor laterality, *n* (%)	Right colon	43 (86.0)	0.559	43 (86.0)	0.139
	Left colon	66 (90.4)		70 (95.9)	
	Rectum	142 (84.5)		150 (89.3)	
Tumor grade, *n* (%)	I	198 (87.6)	**<0.001**	208 (92.0)	**0.001**
	II	40 (83.3)		41 (85.4)	
	III	4 (50.0)		5 (62.5)	
Number of extracted lymph nodes, *n* (%)	<5	89 (86.4)	0.584	98 (97.5)	0.496
	5–12	65 (92.9)		68 (90.6)	
	>12	94 (89.5)		97 (89.8)	
Perineural invasion, *n* (%)	Yes	5 (83.3)	0.899	5 (83.3)	0.667
	No	246 (86.3)		258 (90.5)	
Histologic type, *n* (%)	Mucinous	18 (85.7)	0.718	19 (90.5)	0.178
	Non-mucinous	112 (91.1)		120 (97.6)	
T level, *n* (%)	T1	38 (88.4)	0.438	39 (90.7)	0.790
	T2	200 (85.1)		211 (89.8)	
Obstruction, *n* (%)	Yes	6 (85.7)	0.800	5 (71.4)	0.184
	No	245 (86.3)		258 (90.8)	
Perforation, *n* (%)	Yes	7 (87.5)	0.885	6 (75.0)	**0.020**
	No	244 (86.2)		257 (90.8)	
Tumor appearance, *n* (%)	Fungative or polypoid	49 (86.0)	0.178	51 (89.5)	0.240
	ulcerative	47 (83.9)		46 (82.1)	
	Ulcerative fungative	12 (75.0)		13 (81.3)	
	Infiltrative	13 (72.2)		14 (77.8)	
Lymphovascular invasion, *n* (%)	Present	13 (72.2)	0.072	15 (83.3)	0.197
	Absent	238 (87.2)		248 (90.8)	

* *P*-value was estimated by the log-rank test for statistical univariate analysis.

Bold values represent significant results (*P* < 0.05).

**Figure 2:**
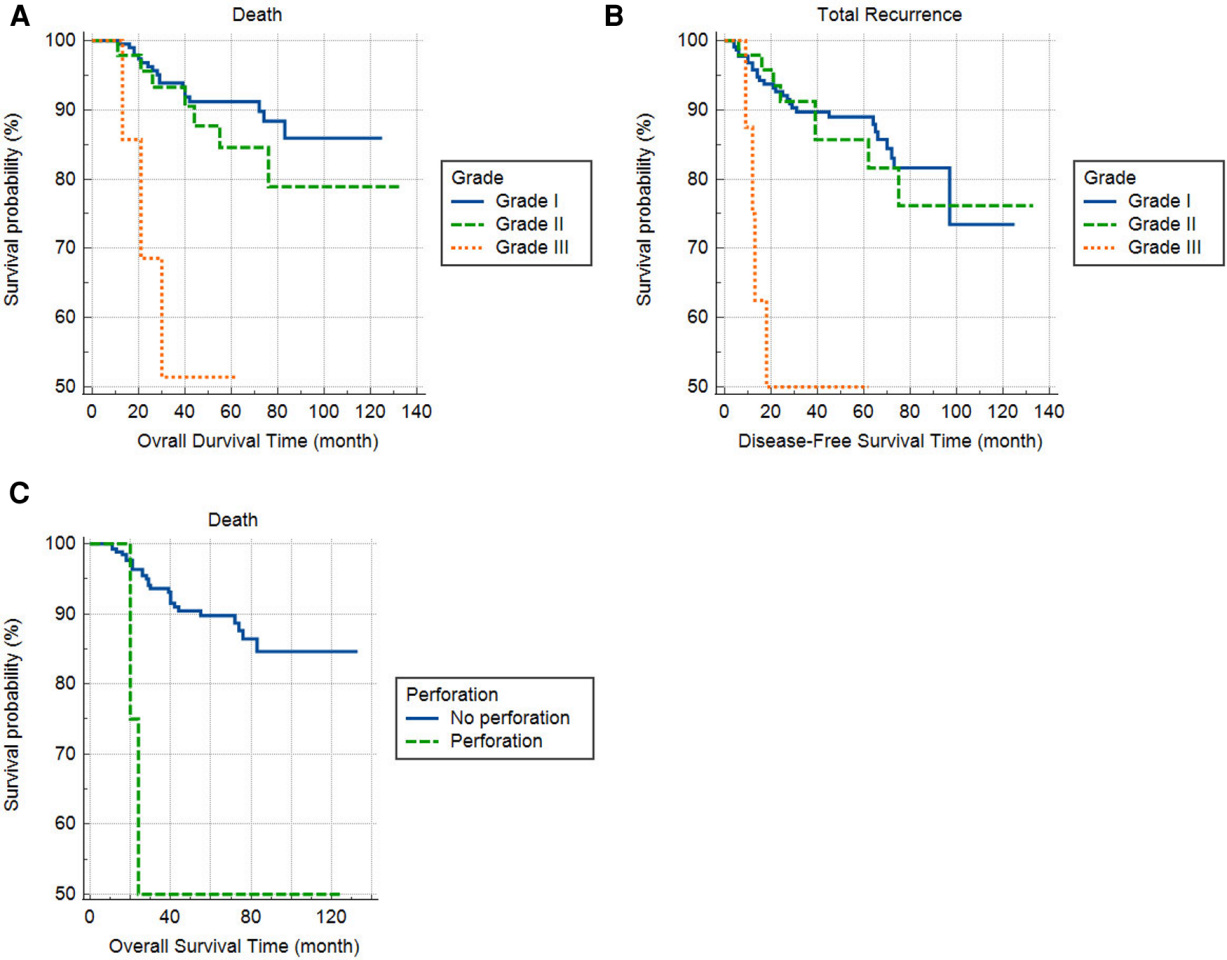
The effect of tumor grade on overall (**A**) and recurrence-free survival (**B**) and perforation on overall survival (**C**).

Cox regression analysis revealed an association between tumor grade III with RFS [hazard ration (HR) III/I = 7.58, 95% confidence interval (CI): 2.58–22.34, *P* < 0.001] and OS (HR III/I = 8.37, 95% CI: 2.42–228.94, *P* = 0.001). Furthermore, patients with perforation had worse survival (HR = 4.68, 95% CI: 1.08–220.26, *P* = 0.039), while other variables were insignificant or lost their significance in multivariable regression analysis (Table 3). The association of tumor grade and perforation with overall and recurrence-free survival is depicted in Figure 2.

**Table 3:** Multivariate Cox regression analysis of prognostic factors for RFS and OS in patients with stage I CRC.

Variable		RFS		OS	
		HR (95% CI)	*P*-value	HR (95% CI)	*P*-value
Tumor grade (vs. grade I)	II	1.18 (0.54–2.58)	0.686	1.45 (0.60–3.50)	0.407
	III	7.58 (2.58–22.34)	<0.001	8.37 (2.42–28.94)	0.001
LVI		2.48 (0.96–6.37)	0.060	–	
Perforation		–		4.68 (1.08–20.26)	0.039

CI, confidence interval; CRC, colorectal cancer; RFS, recurrence–free survival; HR, hazard ratio; LVI, lymphovascular invasion; OS, overall survival.

## 4 Discussion

Rising rates of exposure to risk factors and advancements in the early detection of CRC, particularly through the integration of artificial intelligence (21, 22), are expected to contribute to an increasing trend in CRC incidence in the coming years. Therefore, it is essential to identify factors associated with improved survival across diverse populations in order to identify high-risk individuals and implement tailored management strategies. Novel approaches such as internet-based interventions (23) can be leveraged to optimize clinical outcomes for these individuals.

Our study evaluated all patients with stage I CRC who underwent surgical resection during a nine-year period in terms of demographic and clinicopathological characteristics and their association with patient outcomes (OS and RFS). The overall recurrence rate was higher than that reported by most of the previous reports (4.1%–7.1%) (24–28) but is lower than the study by Patel et al., who reported 16.8% local recurrence (29). This discrepancy in the reported recurrence rates is possibly related to differences in surgical techniques and study designs (time to event, retrospective vs. prospective, sample size, etc.). Furthermore, a missed diagnosis of micrometastasis to lymph nodes (≤2 mm) is also possible (30). Including patients with <12 lymph nodes resected in our study, which can result in under-staging the disease, could also be another reason for the high recurrence rate. The OS and RFS rates in the present study were lower than Lee et al.'s findings (93.5% and 95.7%, respectively) (24) but similar to Teloken et al.'s RFS of 83.2% (28) in patients with stage I CRC. This difference can be related to variations in screening programs and treatment protocols. As mentioned, we have not included patients who underwent neoadjuvant chemotherapy. Furthermore, differences in patient-related and tumor-related factors in the study populations could be another source of variation.

Considering the patients' demographics and their association with patients' outcomes, we observed that men had a higher frequency and mean age. Data from England's national report (31) and other studies on patients with stage I CRC (25, 32) also report a male dominance of stage I CRC, which is in line with the results of the present study. This finding may be attributed to the deprivation of men from the protective effects of estrogen and a more harmful lifestyle involving smoking, alcohol, and unhealthy food (31). However, our results did not show an effect of sex on the OS, RFS, or recurrence rates. Others reported similar results (24, 25, 28, 31), while Patel et al. reported more recurrence in men, possibly related to the greater difficulty of complete resection in men (29). Due to the diversity in treatment protocols and tumor-related factors, the results of studies cannot be easily compared.

Considering patients' age, about 9% of our study sample were young (<40 years), which seems higher than similar reports (32) but is in line with the overall higher frequency of early-onset CRC reported in Iran compared with Western countries (33). The increasing trend in early-onset CRC has been attributed to the greater tendency of this population toward a sedentary and unhealthy lifestyle (19, 34), which calls for greater attention to this age group, who might benefit from particular treatment strategies (35). Nevertheless, we did not observe any effect of age on the OS, RFS, or recurrence, consistent with previous reports (24–26, 29). However, some have reported age as a significant predictor of RFS (HR = 1.05) (28), and others have reported worse prognosis in younger patients with T1 CRC, associated with a higher susceptibility to lymph node and distant metastases (36, 37). One reason for such a variation is differences in considering cancer-related deaths or all-cause deaths when calculating survival.

Among the various tumor-related factors evaluated in the present study, perforation was associated with a 4-fold worse overall survival but had no effect on RFS. There is limited evidence on the impact of perforated CRC on outcomes. Belt et al. reported a higher recurrence rate as well as worse overall survival and disease-free survival in stage I/II colon cancer patients with peri-operative perforation compared to patients without (38). However, in a study on patients with stage I–III rectal cancer, the recurrence rate, but not metastasis or overall survival, was increased after perforation in the multivariable analysis (39). On the other hand, Orive et al. reported that perforation was predictive of early recurrence at T2 but not T1 in patients with colon cancer (40). These discrepancies could be attributed to differences in tumor stages and definitions of perforation across studies (41).

In the present study, a trend towards an increased risk of RFS was observed in patients with positive LVI, although it was not statistically significant. The results of a meta-analysis of 9,881 patients with stage I/II CRC showed HR = 2.15 for OS and 1.73 for disease-free survival (42), emphasizing the prognostic value of LVI, similar to the results of other previous reports (26, 43). Patel et al. reported that the low number of LVI in their study population (14%) was the reason for the lost significance of LVI's association with recurrence in multivariate regression (29). The frequency of LVI was even lower in our study. Therefore, the reasons for the discrepancy in the study results in terms of LVI include the different prevalence of LVI among study populations and differences in the diagnostic methods used for reporting LVI as positive.

Our study revealed an association between elevated rates of distant metastasis and local recurrence with tumor grade. These results align with existing literature concerning individuals diagnosed with early stage CRC (29, 44, 45). As, Li et al., reported that higher tumor grade was associated with an increased risk of distant metastasis in patients diagnosed with stage T1 CRC, with 90% of the study's participants presenting with stage N0 (44). Cox regression analysis also showed that patients with tumor grade III had 7.5-fold higher odds of recurrence and 8.4-fold higher odds of mortality than those with tumor grade I. Although studies on the T1 stage have considered tumor grade as a predictor of OS (37) and recurrence (36, 37), such association was not reported in stage I CRC (24). As T1 can be observed at other stages, more studies with larger sample sizes are required on stage I CRC. Therefore, more studies are required in this regard. All in all, there is a great discrepancy in the results of studies on the factors associated with recurrence and survival in patients with stage I CRC, and differences in the definitions and diagnostic and treatment methods limit comparisons and call for standardized studies in this regard.

The present study had some limitations, including the study's retrospective nature, the limited number of samples with specific events, the short duration of follow-up, and the selection of all samples from one city by census method. However, the surgeons and physicians involved in the study were consistent in their approach and adhered to similar protocols throughout the nine years. A further limitation of the current study is the low number of extracted lymph nodes, which could be ascribed to the early stage of CRC and the small tumor sizes observed in the included patients (46, 47). Several other factors may also affect patient outcomes that were not assessed here, such as tumor budding, patients' education and economic status, and molecular and biological characteristics of tumors. Future prospective studies with adequate lymphadenectomy, standardized surgical procedures, and increased follow-up period are recommended for colon and rectum cancers separately. Furthermore, the implementation of novel diagnostic and staging techniques, including the integration of deep learning algorithms (21, 22), could enhance accuracy and efficiency.

## 5 Conclusion

The present study found that tumor grade III was associated with higher recurrence rates and poorer OS and RFS in patients with stage I CRC. Additionally, perforation was found to be associated with worse OS. Therefore, early diagnosis of high-risk patients through close postoperative follow-up and selection of more aggressive and extensive treatment strategies for this group could improve patients' outcomes.

## Data Availability

The raw data supporting the conclusions of this article will be made available by the authors, without undue reservation.
